# Spermatogenesis-specific Argonautes ALG-3/4 promote LEA-1 expression in oocytes

**DOI:** 10.17912/micropub.biology.002196

**Published:** 2026-08-18

**Authors:** Thomas Liontis, Alla Grishok

**Affiliations:** 1 Department of Biochemistry & Cell Biology, Chobanian & Avedisian School of Medicine, Boston University, 72 East Concord Street, Boston, MA, 02118, USA; 2 Graduate Program in Genetics and Genomics, Chobanian & Avedisian School of Medicine, Boston University, Boston, MA, 02118, USA; 3 Genome Science Institute, Boston University, Boston, MA, 02118, USA

## Abstract

Small interfering RNAs bound to Argonautes ALG-3 and ALG-4 ( ALG-3 /4) in spermatocytes regulate fertility and aging in *C. elegans* . ALG-3 /4 cell non-autonomously repress DAF-18 /PTEN in the oocyte, thereby limiting the activity of DAF-16 /FOXO and longevity. We find that the DAF-16 and ALG-3 /4 target gene *lea-1* is downregulated in precursor germ cells and oocytes of *alg-3 /4* mutants while being upregulated in *age-1 ( hx546 )* PI3K mutants, where DAF-16 is activated. The downregulation of *lea-1* in *alg-3 /4* mutants is inherited, unlike the elevation in DAF-18 levels. We therefore propose that ALG-3 /4 regulate LEA-1 and DAF-18 through distinct non-autonomous mechanisms.

**Figure 1. ALG-3/4 promote lea-1 expression cell non-autonomously in pachytene germ cells and oocytes:** **(A)** Representative images of endogenously-tagged mNeonGreen::3xFLAG::AID:: LEA-1 (mNG:: LEA-1 ) and differential interference contrast (DIC) microscopy of L4 male WT and *alg‑3( tm1155 ); alg‑4( ok1041 )* mutant worms. Yellow arrow indicates spermatocytes. **(B)** Quantification of mean mNG:: LEA-1 in spermatocytes from panel A. **(C)** Representative mNG:: LEA-1 and DIC images of WT, *alg-3 /4* mutants, *age-1 ( hx546 )* , and triple mutant *age-1 ; alg-3 /4* young adult hermaphrodites. Red arrows indicate pachytene-stage germ cells, blue arrows indicate the −1 oocyte. **(D – F)** Quantification of mNG:: LEA-1 in pachytene-stage germ cells indicated in panel C. **(G)** Quantification of mNG:: LEA-1 in −1 oocytes indicated in panel C. **(H)** Mean mNG:: LEA-1 fluorescence in the −1 oocyte of young adult F1 progeny arising from the indicated crosses. *alg-3 /4(-)* : *alg-3 ; alg-4* double mutants. **(I)** Model of the regulation of LEA-1 by IIS and ALG-3 /4. In spermatocytes, ALG-3 /4 bind to siRNAs that target mRNAs, promoting *lea-1* and repressing a subset of *msp* genes (Liontis et al., 2026) while promoting others (Conine et al., 2013). Mutating *alg-3 /4* causes a decrease in *lea-1* mRNA and protein levels in spermatocytes, resulting in decreased LEA-1 protein in the oocyte through an unknown mechanism (arrow with "?"), likely separate from the derepressed MSP activity that results in disrupted EphR ( VAB-1 ) localization and increased DAF-18 levels (Liontis et al., 2026). DAF-18 is a phosphatase that dephosphorylates PIP _3_ . In contrast, DAF-2 promotes the activation of AGE-1 , which phosphorylates PIP _2_ to PIP _3_ , resulting in repressed DAF-16 activity. In *daf-2* or *age-1* mutants, DAF-16 is activated and upregulates *lea-1* mRNA and protein levels. Scale bars: 50 μm.

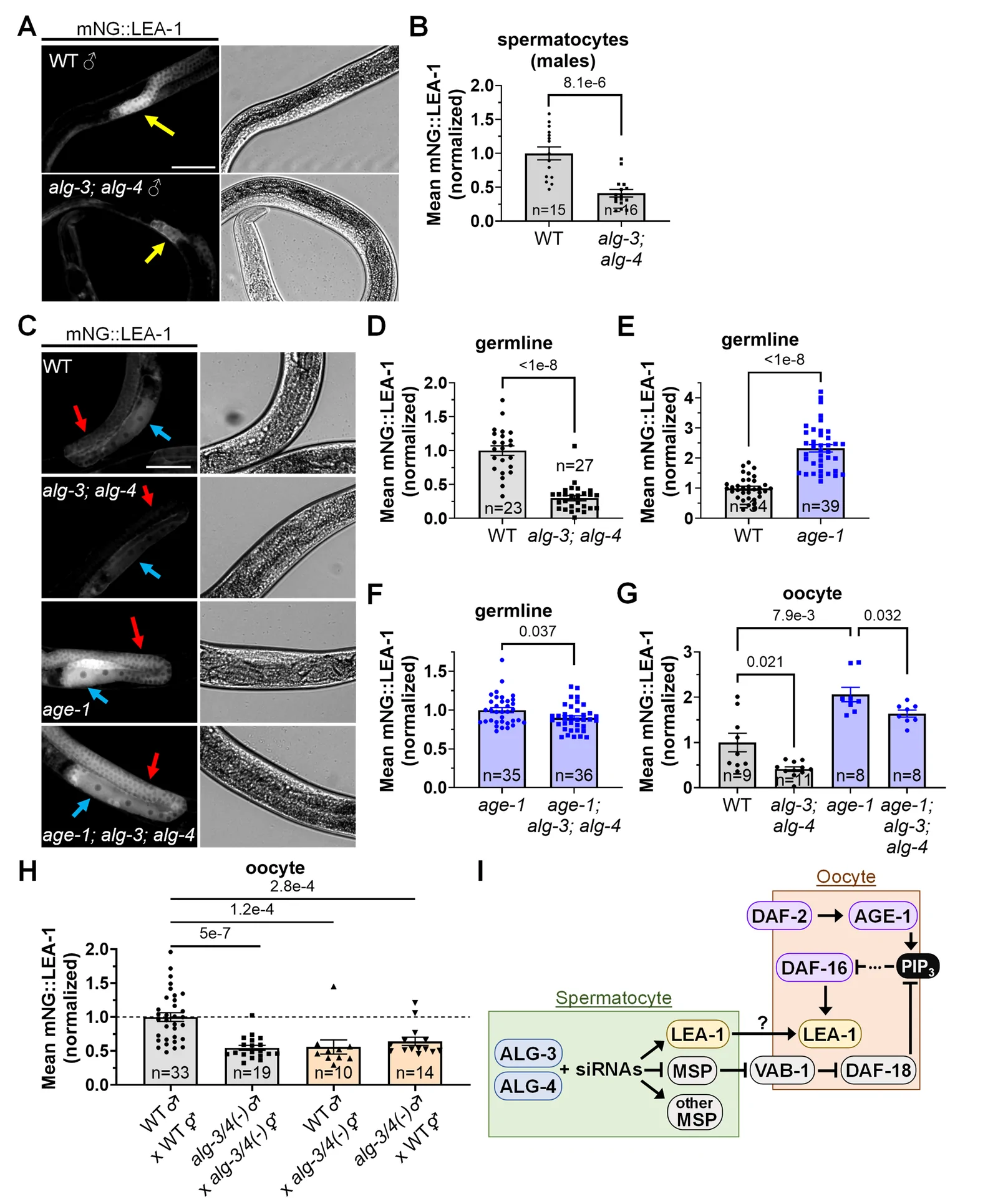



## Description

In *C. elegans* , exogenous and endogenous double-stranded RNAs are recognized and bound by RDE-4 , which directs them to Dicer for cleavage (Boyle et al., 2026; Parker et al., 2006; Tabara et al., 2002; Thivierge et al., 2011). Subsequent processing of these RNAs results in double-stranded primary small interfering RNAs (siRNAs) (Duchaine et al., 2006; Lee et al., 2006; Yigit et al., 2006), which bind to Argonaute proteins. The Argonautes ALG-3 and ALG-4 ( ALG-3 /4) are redundant and expressed in spermatocytes during spermatogenesis (Conine et al., 2010; Han et al., 2009; Vasale et al., 2010). Their disruption causes defects in spermatocyte maturation and sperm function (Conine et al., 2010). ALG-3 /4 can repress or promote the expression of their targets, with the latter process being enhanced at 25 °C (Conine et al., 2010, 2013). One gene with decreased levels of corresponding endo-siRNA, mRNA, and protein in *alg-3 /4* mutant males, i.e. a direct target of ALG-3 /4, is *lea-1* (Conine et al., 2013).

The *lea-1* gene is robustly upregulated in long-lived mutants of the insulin/IGF-1 (IIS) pathway such as the insulin/IGF-1 receptor *daf-2* mutant (Chen et al., 2015) and PI3K catalytic subunit *age-1* mutant (Liontis et al., 2026). As is the case for the lifespan extension of IIS mutants (Kenyon et al., 1993; Klass, 1983; Murakami & Johnson, 1996), the upregulation of *lea-1* in *daf-2* mutants requires the downstream activation of the forkhead box O (FOXO) transcription factor DAF-16 (Chen et al., 2015). However, *lea-1* does not appear to play a role in aging and is dispensable for the longevity of *daf-2* mutants (Hibshman & Goldstein, 2021; Zečić et al., 2022).

We previously found that *alg-3 /4* mutations cause the disruption of Eph receptor (EphR) perimembrane localization and the elevation of PTEN protein levels in the neighboring mature oocyte (Liontis et al., 2026). These effects caused by *alg-3 /4* mutations were associated with the enhancement of the lifespan and healthspan of *age-1 ( hx546 )* mutant hermaphrodites, but not males (Liontis et al., 2026). In addition, paternal sperm carrying the *alg-3 /4* mutations was not sufficient to elevate oocyte PTEN levels in progeny, and paternal sperm carrying wild-type *alg* -3/4 was also not sufficient to rescue the particularly low brood size of *age-1 ; alg-3 /4* mutants (Liontis et al., 2026). Taken together, our previous findings are consistent with spermatogenically-expressed ALG-3 /4 regulating the oocyte in the same generation in a cell non-autonomous manner.

To further understand the spatial dynamics of genes regulated by ALG-3 /4, we aimed here to measure the non-autonomous effects of ALG-3 /4 on the expression of their target gene LEA-1 . In addition, we were interested in assessing the interaction between the ALG-3 /4 and IIS pathways in regulating a common gene target. Although LEA-1 does not appear to play a role in aging, it uniquely satisfies the conditions of being expressed in various tissues, including spermatocytes and oocytes, being regulated by both ALG-3 /4 and IIS, and having a readily available reporter strain, LP858 , where its coding region was endogenously tagged with a fluorescent protein using CRISPR (mNeonGreen:: LEA-1 ) (Hibshman & Goldstein, 2021).

We confirmed that ALG-3 /4 promote LEA-1 protein levels in males by observing a strong decrease in mNeonGreen:: LEA-1 levels in the spermatocytes of *alg-3 ( tm1155 ); alg-4 ( ok1041 )* mutant males **( Figure 1A, B)** . We then assessed LEA-1 levels in hermaphrodites **( Figure 1C –G)** . Intriguingly, we found a strong downregulation of mNeonGreen:: LEA-1 in the pachytene stage germ cells and proximal (−1) oocyte of *alg-3 /4* mutant hermaphrodites **( Figure 1C, D, G)** . Given that ALG-3 and ALG-4 are specifically expressed in spermatocytes (Charlesworth et al., 2021; Conine et al., 2010), this finding is consistent with non-autonomous effects of ALG-3 /4. Consistent with *lea-1* being the most statistically significantly upregulated gene in *age-1 ( hx546 )* mutants according to our RNA-sequencing data (Liontis et al., 2026), mNeonGreen:: LEA-1 levels were considerably increased throughout the germline of *age-1* mutant hermaphrodites **( Figure 1C, E, G)** . In these *age-1* mutants, the *alg-3 /4* mutations caused a statistically significant but markedly attenuated reduction in mNeonGreen:: LEA-1 levels **( Figure 1C, F, G)** . In other words, the *age-1 ( hx546 )* background largely (but not entirely) suppressed the effect of *alg-3 /4* mutations in reducing LEA-1 levels in pachytene germ cells and the proximal oocyte.

Finally, we crossed wild-type (WT) and *alg-3 /4* mutant worms to observe the effects of maternal or paternal loss of *alg-3 /4* on the LEA-1 levels of progeny. Cross-progeny of *alg-3 /4* mutant parents displayed significantly decreased mNeonGreen:: LEA-1 levels in their proximal oocytes, compared to cross-progeny of WT worms, as expected from these control conditions **( Figure 1H )** . Surprisingly, progeny of WT males crossed to *alg-3 /4* mutant hermaphrodites, like those of *alg-3 /4* mutant males crossed to WT hermaphrodites, also exhibited a similar decrease in mNeonGreen:: LEA-1 levels in their proximal oocyte **( Figure 1H )** . This suggests that the process resulting in the downregulation of *lea-1* in *alg-3 /4* mutants can be inherited both maternally and paternally. Given that these progeny animals are heterozygous for the *alg-3 /4* mutations, an alternative explanation is that *alg-3 /4* are haploinsufficient, at least with respect to their regulation of *lea-1* . This interpretation is unlikely because *alg-3 /4* heterozygotes do not phenocopy the sterility at 25 °C of *alg-3 /4* homozygous mutants (Conine et al., 2010, 2013). In addition, it was shown that male *alg-3 /4* heterozygotes do not display a decrease in *lea-1* pre-mRNA levels compared to WT, unless they descend directly from a homozygous *alg-3 /4* mutant parent (Conine et al., 2013). Overall, although it is unclear how ALG-3 /4 promote LEA-1 expression outside of spermatocytes, ALG-3 /4 must be functional in both the father and hermaphrodite mother to sustain LEA-1 expression in the oocyte of their progeny.

In this work, we found that ALG-3 /4 not only promotes LEA-1 levels in male spermatocytes but also in the oocyte and precursor germ cells of hermaphrodites **( Figure 1I )** . Given that LEA-1 is repressed by IIS, this is a system where ALG-3 /4 and AGE-1 have opposite effects on gene expression. In contrast, ALG-3 /4 and AGE-1 both inhibit DAF-16 activity in their regulation of lifespan (Liontis et al., 2026). The downregulation of LEA-1 despite the increased activation of DAF-16 in *alg-3 /4* mutants is paradoxical, suggesting that there is likely an additional signaling pathway regulated by ALG-3 /4, separate from the MSP/EphR/PTEN system (Liontis et al., 2026), at play **( Figure 1I )** . This is further supported by the fact that the increase in DAF-18 (PTEN) levels in the oocyte of *alg-3 /4* mutants could not be inherited (Liontis et al., 2026), in contrast to the decrease in LEA-1 levels **( Figure 1H )** . In both cases, the dysregulation of these genes in *alg-3 /4* mutants cannot be simply reasoned as disruptions of ALG-3 /4 in paternal sperm. Instead, our results suggest that impairments causing LEA-1 downregulation in *alg-3 /4* mutant males and hermaphrodites are likely inherited throughout development and adulthood, whereas those causing DAF-18 elevation are present only in the generation where both copies of *alg-3 /4* are mutated. It is therefore likely that DAF-18 and LEA-1 are regulated by separate pathways controlled by ALG-3 /4. Overall, our studies of ALG-3 /4 reveal multiple cell non-autonomous gene regulatory mechanisms.

## Methods

***C. elegans* strains and maintenance**

Worms were cultured and assayed at 20 °C on solid nematode growth media (NGM) seeded with *E. coli* OP50 . The mNG:: LEA-1 strain LP858 was outcrossed once to eliminate an unexpected dumpy (dpy)-like phenotype. The resulting strain was then crossed to the *age-1* and *alg-3 ; alg-4* mutant strains, which have already been outcrossed six times.

**Fluorescence microscopy**

Compound microscopy was performed using the Zeiss AxioImager Z1. Worms were mounted on 2% agarose pads, paralyzed with 10–20 mM levamisole in M9 buffer, placed under a thin glass coverslip, and immediately imaged. A constant exposure time was always used for images shown in the same figure panel. In addition, controls were always present on the exact same agarose pad as experimental animals (e.g., mutants). The ImageJ (Fiji) software was used for quantification of mNeonGreen:: LEA-1 fluorescence and this analysis was performed while blinded to the strains' genotype. Mean fluorescence was normalized to controls in each independent experimental replicate, and results were pooled to produce the graphs shown in the figure. Random counterbalancing of the order in which strains were imaged was done between each independent replicate. Regions of interest were delimited manually: spermatocytes for L4 males; pachytene-stage germ cells in the proximal Rachis region and the proximal −1 oocyte's cytoplasmic region for young Day 1 adult hermaphrodites. At this stage, oocytes were large and mature but no more than 1 embryo was present.

**Statistical analyses and graphs**

Statistics were analyzed as was done previously (Liontis et al., 2026). Briefly, biological replicates (n) represent the number of animals, whereas independent experimental replicates (N) represent the number of independent experiments started on a different day with independent populations of animals. When comparing means, the F-test for variance and Anderson-Darling, D'Agostino-Pearson, Shapiro-Wilk, or Kolmogorov-Smirnov tests for normality were computed first. The resulting appropriate unpaired Student's t-test (equal vs. unequal variance) or Mann-Whitney test (nonparametric) was then used to assess a two-tailed significant difference between two groups. Tests were computed and graphs were generated using GraphPad Prism 10.

Error bars represent the standard error of the mean.

**Parental effects on LEA-1**

All strains were homozygous for mNeonGreen:: LEA-1 . Three males and one hermaphrodite at the L3 – L4 stage were transferred to each plate, with several plates for each cross. Hermaphrodites were considered to have mated when a significant number of progeny (F1) were males (up to 50%). F1 late L4 hermaphrodites that were identified as cross-progeny, i.e. not older than their sibling F1 males, were transferred to a new plate to prevent new crossing as a confounding factor. These F1 animals were imaged 7 hours after being transferred, at which point they reached young adulthood.

## Reagents

**Table:** 

Strain name	Genotype	Source
N2	*C. elegans* wild type	*Caenorhabditis* Genetics Center
TJ1052	*age-1 ( hx546 ) II *	*Caenorhabditis* Genetics Center
WM300	*alg-4 ( ok1041 ) III; alg-3 ( tm1155 ) IV *	*Caenorhabditis* Genetics Center
LP858	lea-1 ( cp431 [mNG::3x FLAG::AID*:: lea-1 ]) V	*Caenorhabditis* Genetics Center
AGK1001	*alg-4 ( ok1041 ) III; alg-3 ( tm1155 ) IV; lea-1 ( cp431 ) V *	AGK lab
AGK1003	*age-1 ( hx546 ) II; lea-1 ( cp431 ) V *	AGK lab
AGK1000	*age-1 ( hx546 ) II; alg-4 ( ok1041 ) III; alg-3 ( tm1155 ) IV; lea-1 ( cp431 ) V *	AGK lab
OP50	* E. coli OP50*	*Caenorhabditis* Genetics Center
